# Clinical Applications of Circulating Tumour DNA in Pancreatic Adenocarcinoma

**DOI:** 10.3390/jpm9030037

**Published:** 2019-07-18

**Authors:** Matthew Loft, Belinda Lee, Jeanne Tie, Peter Gibbs

**Affiliations:** 1 Systems Biology and Personalised Medicine Division, Walter and Eliza Hall Institute of Medical Research, Parkville 3050, Australia; 2 Department of Medical Biology, The University of Melbourne, Parkville 3010, Australia; 3 Department of Medical Oncology, Western Health, Footscray 3011, Australia; 4 Department of Medical Oncology, Peter MacCallum Cancer Centre, Melbourne 3000, Australia

**Keywords:** pancreatic cancer, circulating tumour DNA (ctDNA), biomarkers, liquid biopsy, personalised oncology, targeted therapy, intratumoural heterogeneity, molecular resistance

## Abstract

Pancreatic adenocarcinoma remains one of the most aggressive cancers with an ongoing dismal survival rate despite some recent advances in treatment options. This is largely due to the typically late presentation and limited effective therapeutic options in advanced disease. There are numerous circulating biomarkers that have potential clinical application as tumour markers, including circulating tumour DNA (ctDNA), circulating tumour cells (CTCs), cell-free RNA (cfRNA), exosomes and circulating tumour proteins. This review will focus on the development of ctDNA as a non-invasive liquid biopsy, with its high sensitivity and specificity having potential clinical applications in pancreatic cancer. These include a role in screening, prognostication via the detection of minimal residual disease, early detection of recurrence, and for patients with advanced disease; tumour genotyping and monitoring treatment response. Prospective randomised adjuvant clinical trials are currently underway, exploring the impact of ctDNA-guided adjuvant therapy decisions. In this review, we provide perspectives on the current literature and considerations of future directions.

## 1. Introduction

Although not a common cancer, pancreatic ductal adenocarcinoma (PDAC) is the fourth leading cause of cancer-related death in developed countries and the seventh leading cause of cancer-related death worldwide [1,2]. Pancreatic cancer is projected to be the second leading cause of cancer-related death by 2030 [3]. PDAC is reported to have the lowest 5-year survival of all cancers (9%), which is in part due to the limited therapeutic advancements over the last 10 years, compared to other cancers [4]. PDAC comprises 85% of all primary pancreatic cancers, and although potentially curative via surgical resection, 80–90% of patients will present with unresectable local or distant metastatic disease [5]. Even in the small number of patients with local disease at diagnosis, the 5-year survival rate is only 32% [5].

Studies utilising exome sequencing to assess clonal evolution of pancreatic cancer indicate a long window of opportunity may exist for early detection, maybe even in the realm of 20 years before any symptoms occur [6,7]. However, with no sensitive and specific biomarker, there is currently no role for screening the general population, and the continued inability to detect tumours at an early stage continues to compromise outcomes. Fortunately, in the last decade, some progress has been made in the treatment of advanced disease, with FOLFIRINOX chemotherapy (a combination of oxaliplatin, irinotecan and 5-fluorouracil) extending median survival to 11 months [8], albeit with significant toxicity. The addition of nab-paclitaxel to gemcitabine has also been shown to prolong overall survival (OS) in advanced disease [9]. Although these advances are significant, treatment is always with palliative intent and there are no long-term survivors.

Circulating cell-free deoxyribonucleic acid (cfDNA) was initially discovered by Mandel and Metais in 1948 [10], but it was not until 1977 that cfDNA was found to be at a higher concentration in the peripheral circulation of cancer patients [11]. cfDNA is the term for genetic fragments in the form of nucleic acid chains that can be found in multiple bodily fluids including blood, stool, urine and saliva. In healthy individuals, the majority of cfDNA originates from haematopoietic cells. Circulating tumour DNA (ctDNA) refers to the proportion of cfDNA that is derived from tumour cells in patients with malignancy. The exact mechanism by which ctDNA enters the bloodstream remains unclear, but tumour DNA is postulated to be released from tumour cells via apoptosis or necrosis [12]. Once in the circulation, ctDNA is swiftly cleared, with a half-life of around 2 hours [13,14], suggesting prospects as a close to real-time dynamic biological marker of tumour burden. Given that tumour-specific genetic abnormalities (point mutations, chromosomal translocation, methylation changes) can be detected in the circulation, the exquisite specificity of ctDNA presents another desirable feature as a biomarker.

ctDNA has been restricted in its clinical application until recently due to the incapacity to reliably detect and compute the extremely low frequency of DNA variants that may be present in a blood sample, particularly in the context of screening of minimal residual disease. In an attempt to increase the sensitivity and specificity of ctDNA detection, specialised methods for analysis have been developed, ranging from technologies used for single mutation to entire genome analysis. However, ctDNA concentrations as low as <0.01% of the total cfDNA can now be detected, with ctDNA concentrations correlating with tumour burden [15,16]. The single mutation or limited mutation panel strategy utilises detection of individual point mutations through polymerase chain reaction (PCR)-based assays, such as droplet digital PCR (ddPCR) and beads, emulsion, amplification and magnetics (BEAMing). These techniques offer greater sensitivity than non-targeted approaches, but necessitate prior knowledge of mutation information [17]. Non-targeted methodologies such as whole genome sequencing (WGS) using next generation sequencing (NGS) do not require prior tumour mutation information [18], but do depend on higher ctDNA concentrations and have a lower sensitivity [16]. A sensitivity of ctDNA detection approaching that of PCR-based approaches has recently been achieved utilising target sequencing modified with molecular barcoding to reduce background PCR error rates [17,19,20]. An in-depth review of ctDNA analysis methodologies is beyond the scope of this article. Readers are encouraged to refer to recent reviews [21,22,23].

The lack of standardised guidelines on the use of ctDNA collection and analysis currently presents a clinical hurdle to overcome. As technology improves in assay development and costs decrease, the ability to reliably detect low frequency DNA variants in a blood sample will improve further, facilitating clinical realization.

This review will focus on the evidence surrounding the clinical application of ctDNA in PDAC, given it is by far the most common form of pancreatic cancer. Further references to pancreatic cancer in this review will hence pertain to PDAC only.

## 2. Current Standard of Care for the Diagnosis of Pancreatic Cancer

Diagnosing pancreatic cancer at an early stage is challenging as patients typically only have symptoms when more advanced disease is present and symptoms are often non-specific. However, in the absence of a good screening test, routine screening for asymptomatic individuals is not recommended [24]. Even for high risk patients, such as those with a strong family history of pancreatic cancer, there is no standardised approach to screening.

Currently, the only biomarker used routinely in the management of PDAC post diagnosis is carbohydrate antigen 19-9 (CA 19-9), a sialylated Lewis A blood group antigen [24]. CA 19-9 has an approximate sensitivity of 80% and specificity of 85% in symptomatic patients, but is unreliable as a screening marker due to its low positive predictive value [24]. Of note, Lewis antigen-negative individuals may have undetectable CA 19-9 levels, and elevated CA 19-9 levels can occur in the setting of benign conditions, including cholangitis, biliary inflammation and biliary obstruction [24], severely limiting its utility in the diagnostic setting. Post diagnosis CA 19-9 has demonstrated value in prognosis, higher levels being associated with poorer overall survival and in predicting response to chemotherapy and is clinically used in these contexts [25].

The diagnosis and staging of pancreatic cancer is dependent on imaging (computed tomography (CT), magnetic resonance imaging (MRI)), invasive endoscopic techniques (ultrasound endoscopy fine needle aspiration, endoscopic retrograde cholangiopancreatography) and exploratory laparoscopy. Multi-detector computed tomography (MDCT) is the first line imaging modality of choice for suspected pancreatic cancer, followed by MRI for equivocal cases or in the setting of a contraindication to CT [26]. The sensitivity for CT and MRI ranges from 76% to 96% and 83% to 93.5% respectively, and is highly dependent on the size of the lesion [26]. 

Current diagnostic techniques are far from adequate for early detection of pancreatic cancer. With the goal of developing improved strategies to diagnose pancreatic cancer at earlier stages, much focus has been placed on blood biomarkers including “liquid biopsies” such as ctDNA. 

## 3. Clinical Applications of ctDNA in Pancreatic Cancer

### 3.1. Screening

Pancreatic cancer is an aggressive disease, but studies have shown there is an approximate 20-year window in which to detect pancreatic cancer before any symptoms occur [6,7]. This finding has the potential to positively impact on patient survival through earlier diagnosis. Given the current lack of effective clinical screening in early disease, liquid biopsies have been pursued as a way to overcome this dilemma. Unfortunately thus far, the low level of ctDNA in early stage pancreatic cancer has presented a barrier to its use in diagnosis, with advances in ctDNA detection technology yet to accurately detect minute amounts. 

Bettegowda et al. [15] analysed ctDNA from 640 patients with various cancer types and stages and found that ctDNA was detected in 82% of metastatic solid tumours outside of the brain, and approximately 50% of early stage cancers, with detection rates varying with specific tumour types. Specifically, for 155 patients with pancreatic cancer, ctDNA was detected in 48% of localised disease (stage I to III), and approximately 90% in metastatic (stage IV) disease. Two smaller studies have found very similar results. Sausen et al. [27] analysed 51 patients, detecting ctDNA in 43% at the time of diagnosis, with a specificity of >99.9%. Sefrioui et al. [28] compared the diagnostic value of CA19.9, ctDNA and circulating tumour cells (CTCs) in 68 patients. They detected ctDNA in 43% of patients with localised disease and 90% of patients with metastatic disease. ctDNA positivity was also shown to correlate with increased tumour burden and decreased OS in early stages of pancreatic cancer.

In an attempt to increase sensitivity, Cohen et al. [29] assessed the use of KRAS mutations in ctDNA in conjunction with elevated proteins in a study of 221 patients with only resectable pancreatic cancer (Stage I or II). This combination test increased the sensitivity to 64%, maintaining a very high specificity of 99.5% (only one false positive in 182 healthy control patients).

The uncertainty regarding the potential presence of KRAS mutations in benign conditions such as chronic pancreatitis should also be highlighted. Mulcahy et al. [30] detected KRAS mutations in four patients with chronic pancreatitis, all of whom progressed to develop pancreatic cancer. These findings were not reproduced in a larger study which found KRAS mutations in 4 out of 31 patients with chronic pancreatitis, none of whom developed pancreatic cancer within 36 months of follow-up [31]. Kinugasa et al. [32] detected KRAS mutations in ctDNA in 5% of healthy control patients and 20% of those with chronic pancreatitis. Several studies have not detected KRAS mutations in the ctDNA patients with chronic pancreatitis [33,34,35]. As many of the above studies used older techniques, the need for standardised ctDNA measurement and larger prospective studies is paramount to further assess the specificity of KRAS mutation analysis to enable the use of ctDNA in clinical practice. It is worthwhile to note that recent studies have shown that ctDNA methylation analysis has the potential to delineate pancreatic cancer from chronic pancreatitis, providing another possible avenue for diagnostic markers [36,37,38,39,40]. Gene panels comprising abnormalities in methylation have the potential to aid in the diagnosis of pancreatic cancer, but further validation is needed to establish a clinically useful panel.

As a standalone test, ctDNA currently remains restricted in its use in diagnosis due to the low sensitivity and the lack of prospective studies demonstrating the benefit of ctDNA-based screening, the most promising strategy being combining ctDNA measurement with protein markers to achieve greater sensitivity, potentially while maintaining a high specificity.

### 3.2. Tumour Genotyping

Histologic confirmation is required to firmly establish a diagnosis of pancreatic cancer. However, in the context of high clinical suspicion, a non-diagnostic biopsy should not delay resection [24]. Biopsy is typically undertaken via endoscopic ultrasound-guided fine needle aspiration (EUS-FNA). EUS-FNA can be challenging due to anatomical variation and does necessitate sedation. Haemorrhage, pancreatitis and bowel perforation are potential complications. The small risk of intraperitoneal or biopsy needle tract tumour seeding precludes the use of percutaneous biopsy in the initial diagnosis of a patient with a potentially resectable pancreatic mass. Percutaneous biopsies are also associated with a greater bleeding and infection risk [24].

The molecular and genetic landscape of pancreatic cancer is innately complex and heterogeneous, with alterations in approximately 60 genes reported [41,42]. Most commonly, mutations occur in KRAS, CDKN2A, TP53 and SMAD4, with some of these being candidates for targeted therapy [43]. KRAS mutations are the most commonly used targets for ctDNA analysis as they are present in 90–95% of pancreatic cancer [44]. Multiple studies have established that ctDNA profiling can accurately reproduce genomic testing of tumour tissue across different cancers [45,46,47,48].

In 2004, Uemura et al. [49] detected KRAS mutations in 26 of 28 (93%) pancreatic cancers, finding the same mutations in 9 of 26 (35%) in their ctDNA using a mutation-specific assay. However, more recent studies have shown much higher concordance rates between ctDNA and tissue mutation status (75–90%) [15,27,32,50]. A recent prospective cohort study with ctDNA analysis by Lee et al. [51] demonstrated KRAS mutations in 90% of patients enrolled (38/42), achieving 100% concordance on analysis of plasma ctDNA with the same specific KRAS marker in the tumour tissue from each patient. Zill et al. [46] also demonstrated high diagnostic accuracy of cfDNA, with a sensitivity of 97.7% and specificity of 100% when prospectively analysing 26 patients with tumour sequencing. Of note, tumour sequencing failed in nine patients (35%), further highlighting the need for a non-invasive measure where tissue sampling is unsafe, impractical or unsuccessful.

Tumour tissue sampling is currently limited by the need for repeat biopsies in the setting of false negatives, inadequate tumour sample, treatment monitoring, real-time assessment of metastatic disease, disease progression and evaluation of tumour heterogeneity. These limitations coupled with the inability to perform repeat biopsies considerably limits the use of personalized therapies in pancreatic cancer, a niche that liquid biopsies have the potential to fill.

## 4. The Prognostic and Predictive Role of ctDNA

### 4.1. Resectable Pancreatic Cancer and Post-Operative Monitoring

The management of early stage pancreatic cancer is evolving, with consideration of neoadjuvant therapy with either chemotherapy alone [24] or in combination with radiation therapy [52,53,54] and multiple trials exploring the role of adjuvant chemotherapy. The current standard of care in patients fit for treatment following initial surgery is FOLFIRINOX [8]. For less fit patients, options include the combination of gemcitabine and capecitabine [55] or gemcitabine alone [56,57]. This range of treatment options defines the potential utility of blood biomarkers such as ctDNA to improve treatment selection for patients with early stage pancreatic cancer.

Sausen et al. [27] recently demonstrated ctDNA to be a strong prognostic marker. Following disease resection, patients with undetectable ctDNA had a longer DFS (17.6 versus 4.6 months, *p* = 0.03) and a longer OS (32.2 versus 19.3 months *p* = 0.027) than those who were ctDNA positive. Additionally, recurrence was detected 6.5 months earlier utilising serial ctDNA analysis compared with CT imaging alone [27]. Singh et al. [58] also associated higher levels of ctDNA with significantly shorter overall survival and with the presence of vascular encasement and metastatic disease, but found no correlation with qualitative measurement of KRAS mutation status. Yamada et al. [34] associated a poorer prognosis in patients with persistently detectable KRAS ctDNA mutations post-resection, and also a correlation between tumour size and ctDNA detection. Lee et al. [51] also correlated detectable ctDNA post-surgery with significantly poor OS.

Importantly, a meta-analysis of 18 articles with a total of 1243 patients found the presence of ctDNA to be a significant prognostic factor for both progression free and OS in pancreatic cancer [59]. The earlier detection of both recurrence and metastatic disease has the potential to introduce earlier therapy which may improve treatment outcomes and allow more patients with recurrent disease to be actively treated. 

### 4.2. Unresectable Pancreatic Cancer (Locally Advanced and Metastatic)

In the context of pancreatic cancer presenting with metastatic or locally advanced disease, ctDNA analysis has been shown to be an independent prognostic factor and the presence of ctDNA is associated with a shorter DFS and OS [27,60,61,62].

Pietrasz et al. [60] studied 135 patients of all stages and found that the presence of ctDNA was strongly correlated with a shorter overall survival (6.5 versus 19.0 months). In a study of 14 patients, Tjensvoll et al. [61] correlated both radiological changes and CA19-9 level with changes in KRAS mutant ctDNA level, as well as demonstrating the presence of ctDNA to be a significant predictor of both PFS and OS. In 2010, Chen et al. [62] in a study of 91 patients with unresectable disease prior to treatment found KRAS mutations in ctDNA to be an independent negative prognostic factor for survival (OS 3.9 versus 10.2 months, *p* < 0.001). Sausen et al. [27] reaffirmed this finding more recently, demonstrating ctDNA to be an independent prognostic marker of OS in advanced disease, with an OS of 6.5 versus 19.0 months in ctDNA positive and negative patients respectively.

Due to the short half-life of ctDNA, Diehl et al. [14] were able to demonstrate the ability to reliably monitor tumour dynamics in colorectal cancer with serial sampling, an approach that can be applicable to pancreatic cancer. In 2018, Kruger et al. [50] highlighted the value of ctDNA in detecting early response to treatment in the metastatic setting. Rapid decreases in mutant KRAS ctDNA levels were seen early in therapy in some patients and proved to be an early indicator of treatment effect when correlated against radiological imaging. Increases in ctDNA at day 14 correlated with progressive disease on later imaging with a sensitivity of 83% and specificity of 100%. In terms of lead time, ctDNA also showed superiority over biomarkers (both CA19-9 and carcinoembryonic antigen [CEA]). Interestingly, Kruger et al. [50] observed the appearance of a second KRAS mutation during therapy in two patients and the appearance of a KRAS mutation during therapy in one patient who was ctDNA KRAS wild type at treatment initiation.

## 5. Future Directions

For patients with early stage disease, the DYNAMIC-Pancreas study [ACTRN12618000335291] is currently active in Australasia; a multicentre randomised study that will enrol over 400 patients analysing ctDNA informed versus standard of care adjuvant chemotherapy in early stage pancreatic cancer. The study consists of two patient cohorts, with the major focus being on the cohort who received neo-adjuvant therapy prior to curative intent resection. Randomisation will occur after surgery. For those patients randomised to the biomarker-driven arm, ctDNA-negative patients will receive 3 months of adjuvant modified FOLFIRINOX. Those who are ctDNA positive, despite receiving pre-operative FOLFIRINOX therapy, will be switched over to receive 4–6 months of adjuvant gemcitabine-based doublet therapy. In a second separate cohort, this clinical trial will also investigate ctDNA guided adjuvant therapy in patients who undergo immediate resection.

In the metastatic setting, there remain challenges associated with safely obtaining tissue for analysis, where liquid biopsies have a potential role. Concordance studies in pancreatic cancer have supported ctDNA-based tumour genotyping compared with tissue-based genotyping. The ability to noninvasively and reliably predict response to treatment as early as 2 weeks after initiation of therapy is a promising step forward and could become an integral part of patient management. Early information on response to treatment can guide clinicians in real time with regards to futility; avoiding the potential toxicity, inconvenience and cost of treatment with an ineffective therapy. An early switch to an alternative treatment gives the best chance of this being effective or potentially novel treatment concepts could be pursued. While currently not clinically relevant, the potential for ctDNA to detect tumour heterogeneity and the emergence of resistant clones under the selective pressure of a targeted therapy could also guide clinical decision making.

## 6. Conclusions

Limited progress has been made in the diagnosis and management of PDAC. ctDNA analysis shows promise as a biomarker to be used from cancer screening through to the management of advanced disease. As always, this should be proven in prospective clinical trials, with a number of these already underway, with results eagerly awaited.
